# High anti-*Ascaris* seroprevalence in fattening pigs in Sichuan, China, calls for improved management strategies

**DOI:** 10.1186/s13071-020-3935-4

**Published:** 2020-02-12

**Authors:** Youle Zheng, Yue Xie, Peter Geldhof, Johnny Vlaminck, Guangxu Ma, Robin B. Gasser, Tao Wang

**Affiliations:** 1 College of Veterinary Medicine, Sichuan Agricultural University, Chengdu, 611130 Sichuan People’s Republic of China; 2 Laboratory of Parasitology, Department of Virology, Parasitology and Immunology, Faculty of Veterinary Medicine, Ghent University, 9820 Merelbeke, Belgium; 3 Department of Veterinary Biosciences, Melbourne Veterinary School, Faculty of Veterinary and Agricultural Sciences, The University of Melbourne, Parkville, VIC 3010 Australia

**Keywords:** Pig, *Ascaris suum*, Seroprevalence, Serodiagnosis, *As*-Hb-based ELISA

## Abstract

**Background:**

Ascariasis, caused by *Ascaris suum*, is an important soil-transmitted parasitic disease of pigs worldwide. It leads to significant economic losses in the pork industry, as a consequence of low feed conversion efficiency in pigs and liver condemnation at slaughter. Despite ascariasis still being widespread on pig farms in many developing and the industrialised countries, there are surprisingly limited data on porcine ascariasis in China, where nearly half of the world’s total pork is produced.

**Methods:**

In the present study, using the recently developed *A. suum*-haemoglobin (*As-*Hb) antigen-based serological test, we screened 512 individual serum samples from fattening pigs from 13 farms across seven distinct locations of Sichuan Province in China for anti-*Ascaris* antibody.

**Results:**

The prevalence of anti-*Ascaris* antibody ranged from 0% to 100% on the distinct farms, with the mean (overall) seroprevalence being > 60%. There was no significant difference in seroprevalence between the intensive and extensive farms.

**Conclusions:**

To our knowledge, this is the first study to measure anti-*Ascaris* seroprevalence in China. The results of this ‘snapshot’ investigation indicate that *Ascaris* infection in commercial pig farms in Sichuan Province is seriously underestimated, encouraging future, large-scale serological studies to assess the distribution and extent of *Ascaris* exposure and infection in various regions of China and the world.
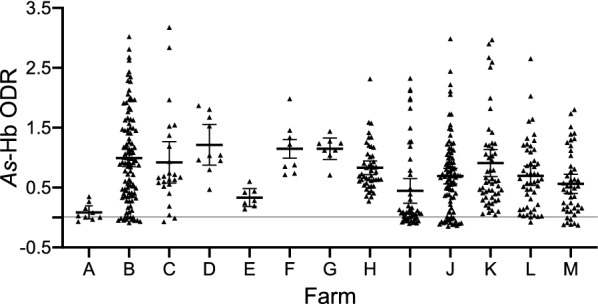

## Background

Ascariasis, caused by *Ascaris suum*, is one of the most important and commonest soil-transmitted parasitic diseases of pigs worldwide. This disease leads to major economic losses to the pork industry as a consequence of reduced weight gain and low feed conversion efficiency in pigs [1] and liver condemnation at slaughter [2]. Currently, losses due to ascariasis have been estimated as at ≥ €4.61 per finisher pig per year [3]. In the field, *A. suum* is often undiagnosed and neglected due to the subclinical nature of infection [4]. Estimates of prevalence and intensity of *Ascaris* infection or exposure on commercial pig farms, achieved using diagnostic tools, support treatment and control programs, and importantly, guide farm management and hygiene practices [5].

A number of diagnostic techniques are available for the detection or assessment of *Ascaris* infection in pigs. They include the qualitative and quantitative flotation of eggs of this nematode from faecal samples; *post-mortem* examination for adult worms or liver lesions; and antibody-based serological tests [6]. Of these diagnostic methods, the recently developed *A. suum* haemoglobin (*As*-Hb) antigen-based enzyme-linked immunosorbent assay (ELISA) test was shown to achieve a higher sensitivity than conventional (qualitative) faecal flotation [7]. Specifically, in well-controlled experimental infection studies, this serodiagnostic tool achieved 99–100% specificity and 90–99.5% sensitivity for detecting the exposure to *A. suum* in fattening pigs [7]. In addition, this serological tool appeared to be able to estimate infection intensity, pig health and farm productivity [8], indicating that it can be used to guide farm management practices and worm control programs.

China provides nearly 50% of the world’s total pork production each year [9]. Although the pork industry contributes substantially to the fast-growing agricultural economy in China, surprisingly limited attention has been paid to infectious diseases including ascariasis. To date, only a small number of coprological surveys of pigs for *A. suum* infection have been conducted on pig farms in the provinces of Hunan (prevalence: 37%; [10]), Chongqing (12%; [11]) and Guangdong (5%; [12]). In the present study, using *As*-Hb antigen-based ELISA, we conducted the first seroprevalence survey to evaluate *A. suum* exposure in fattening pigs of 13 pig farms from seven locations in Sichuan Province, which alone produce 10% of the total amount of pork (per annum) in China [13].

## Methods

### Study population and sample collection

**Fig. 1:**
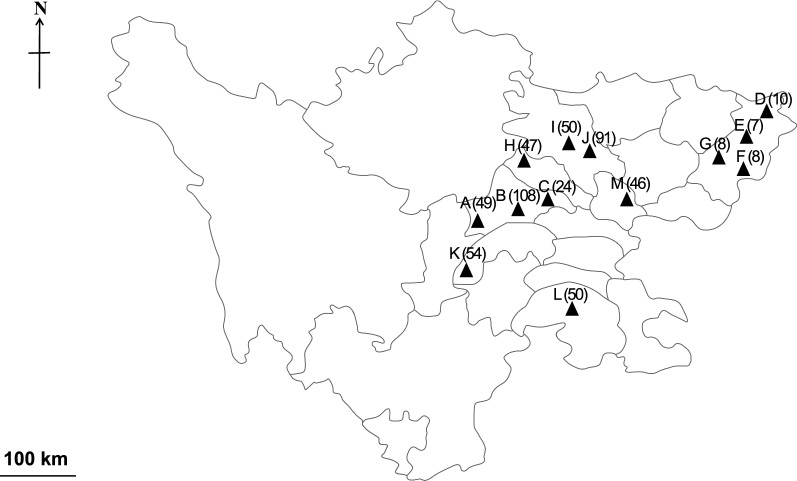
Geographical locations of the 13 studied farms (farms A–M; Table 1) in Sichuan Province, China. Serum samples (numbers in parentheses) were collected from fattening pigs and then tested using the *As*-Hb antigen-based ELISA

From 2015 to 2017, a total of 512 fattening pigs (> 90 kg, 7 months of age) from 11 intensive pig farms and two extensive farms were sampled in Sichuan Province (Fig. 1). In most intensive farms, pigs were kept on fully- or semi-slatted concrete floors, with anthelmintic treatment (e.g. fenbendazole or ivermectin) every three months and high-pressure cleaning of pens after each fattening cycle. On extensive farms, pigs were kept outside without protection from the elements, where they were closely associated with human residences and often fed on household food-waste, with only one anthelmintic treatment (e.g. fenbendazole or ivermectin) during each fattening cycle. Sera were prepared from blood samples following an established protocol [8]. Briefly, blood samples were incubated at 37 °C for 1 h and then refrigerated (4 °C) for 1 h. The sera were then removed from the clot, and centrifugated at 4000×*g* for 10 min at 4 °C. The supernatants were collected and stored at − 20 °C until testing.

### ELISA testing of serum samples

Serum samples were tested as described previously [7]. Briefly, ELISA plates were coated with *As*-Hb (1 µg/ml) in carbonate buffer (pH 9.6) at 4 °C for 18 h, washed three times with phosphate-buffered saline (PBS) plus 0.5% (v/v/) Tween 20 (Sigma-Aldrich, St. Louis, USA) (PBST) and then blocked with 5% (w/v) milk powder in PBS (PBSM) at 4 °C for 2 h. The primary antibody was then added at a dilution of 1/250 in PBST and incubated at 4 °C for 2 h. The plates were washed three times, conjugate (horseradish peroxidase conjugated goat anti-pig IgG, 1/10,000) (Sigma-Aldrich) added and incubated with PBSM at 37 °C for 1 h. Following three final washes with PBST, the substrate o-phenylenediamine 0.1% in citrate buffer (pH 5.0) was added and the optical density (OD) measured at 492 nm. Positive and negative sera were included on each plate. ELISA results were reported as the average optical density ratio (ODR) per farm: OD sample = (OD_Sample_ − OD_Negative Control_)/(OD_Positive Control_ − OD_Negative Control_) and percentage of test-positive animals. The ELISA threshold (between positive and negative) was set at 0.50.

### Statistical analysis

To assess differences in seroprevalence in pigs among different locations and farms, the Chi-square test, one-way analysis of variance (ANOVA) and independent t-test were performed. Probability (*P*) values < 0.05 were considered as statistically significant, and the 95% confidence interval (CI) was used. All analyses were performed using the SPSS Statistics 24 software package (IBM, New York, USA).

## Results and discussion

**Fig. 2:**
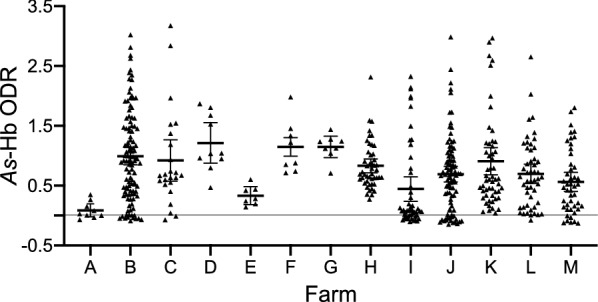
Dot plots displaying optical density ratio (ODR) levels (mean ± standard error, SE) for anti-*Ascaris* antibodies. ODR levels were measured by ELISA in 512 individual serum samples from 13 pig farms (farms A–M; Table 1) in Sichuan Province, China. The horizontal line indicates the cut-off value of ODR = 0.50

Using the *As*-Hb-based ELISA, 512 individual serum samples of fattening pigs from 13 farms from seven distinct locations of Sichuan Province (Fig. 1) were screened for specific anti-*Ascaris* antibody. In total, 60.7% (*n* = 311) of the serum samples were test-positive (OD > 0.5) for anti-*Ascaris* antibodies, with seroprevalence ranging from 14.3% to 100% on 12 fattening farms (Fig. 2; Table 1). Seropositive samples were detected in all locations studied in Sichuan Province. Farm A from Chengdu was the only farm on which no test-positive sera were detected, which is likely due to the relatively small sample size (*n* = 9) tested. Of the eight farms with relatively large sample sizes (*n* > 20), Farm C from the same location as Farm A (i.e. Chengdu) showed the highest seroprevalence (79.2%; 19/24; average ODR of 0.92), whereas Farm I from Mianyang had the lowest prevalence (26.0%; 13/50; average ODR of 0.44). A comparison with findings from previous reports in China showed that the overall *Ascaris* exposure rate in fattening pigs in Sichuan Province (i.e. 60.7%; 311/512; average ODR of 0.78) was markedly higher than that of any other reported regions, such as the provinces Hunan (26.1%; *df* = 1; *P* < 0.001; [10]), Chongqing (12.2%; *df* = 1; *P* < 0.001; [11]) and Guangdong (5.2%; *df* = 1; *P* < 0.001; [12]). Notably, all previous investigations were based on the examination of faecal samples for *Ascaris* eggs, which might have easily underestimated levels of exposure, especially linked to infections with larvae [6, 14]. Although results obtained using the present antigen-based ELISA test indicated a high exposure to *A. suum* on the pig farms studied in Sichuan Province and might be representative, it is possible that the limited number of farms studied and the percentages of animals tested per farm (ranging from 0.23% to 36%; Table 1) might have led to a bias in seroprevalence recorded on these farms. Thus, in the future, large-scale studies should be conducted to establish, compare and contrast anti-*A. suum* seroprevalences in fattening pigs within and among other provinces.

**Table 1:** Summary of anti-*Ascaris* serum antibody prevalence in pigs from thirteen farms in Sichuan, China (cf. Fig. 1)

Location	Farm	No. of samples tested	No. of samples test-positive for anti-*Ascaris* antibody (%)	Average ODR ± SD	95% CI	Total no. of pigs per farm^a^
Chengdu	Farm A (intensive)	9	0 (0.0)	0.09 ± 0.14	− 0.02–0.2	1600–2000
	Farm B (intensive)	108	73 (67.6)	0.99 ± 0.76	0.85–1.13	5000–6000
	Farm C (intensive)	24	19 (79.2)	0.92 ± 0.81	0.58–1.26	3500–3800
Dazhou	Farm D (intensive)	10	9 (90.0)	1.21 ± 0.47	0.87–1.55	800–1200
	Farm E (intensive)	7	1 (14.3)	0.33 ± 0.16	0.18–0.48	1000–2000
	Farm F (intensive)	8	8 (100.0)	1.15 ± 0.44	0.78–1.52	3500–4700
	Farm G (intensive)	8	8 (100.0)	1.15 ± 0.21	0.98–1.33	1100–1200
Deyang	Farm H (intensive)	47	40 (85.1)	0.83 ± 0.39	0.72–0.94	10,000–11,000
Mianyang	Farm I (intensive)	50	13 (26.0)	0.44 ± 0.72	0.24–0.64	11,000–13,000
	Farm J (intensive)	91	56 (61.5)	0.69 ± 0.63	0.56–0.82	10,000–12,000
Ya’an	Farm K (extensive)	54	31 (57.4)	0.91 ± 0.83	0.69–1.13	120–150
Yibing	Farm L (intensive)	50	31 (62.0)	0.70 ± 0.57	0.54–0.86	5000–7000
Suining	Farm M (extensive)	46	22 (47.8)	0.56 ± 0.54	0.40–0.72	200–300
Total		512	311 (60.7)	0.78 ± 0.69	0.72–0.84	

^a^As an all-in/all-out production system was not strictly followed during the fattening cycle on the farms studied here, the approximate range of the total number of pigs on individual farms during fattening cycles is indicated

*Abbreviations*: ODR, optical density ratio; SD, standard deviation; CI, confidence interval

It is generally believed that a better housing system and more regular anthelmintic treatment in modern intensive farms will likely reduce the levels of *Ascaris* infection [15]. This is supported by the previous coprological-based studies in other areas of China as well as in other countries [12, 16], where *A. suum* burden in intensive pig facilities was found to be lower than that on extensive farms. Interestingly, the present study indicates that major *A. suum* exposure can occur in both intensive (62.6%, 258/412) and extensive (53.0%, 53/100) farms in Sichuan Province, with no significant difference between them (*χ*^2^ = 3.124, *df* = 1, *P* = 0.077). Although this finding is challenging to explain, it might relate to the management practices used on the farms studied. For most farms, pigs were imported from local piglet producers, where the management and hygiene conditions were different. In addition, the all-in/all-out production system was not strictly followed in the fattening round on the intensive farms studied (TW, unpublished information). Following initial *Ascaris* egg-contamination, the high stocking density in intensive farms likely led to increased transmission of *A. suum*. It is also worth noting that anthelmintic treatment of pigs on these farms was performed every three months on a regular basis. Given the prepatent period of the life-cycle of *Ascaris* (~42 days; [15]), such a deworming program might not be effective at controlling adult worm burdens and reducing egg contamination in the environment, whereas a six-weekly deworming programme has proven to be efficacious for the control the *Ascaris* infection in intensive piggeries, and was shown also to improve the performance parameters (e.g. feed conversion ratio and average daily growth) in pigs [17]. Preferably, such an efficient deworming program should be introduced on ‘pig fattening’ farms dealing with the challenges of a high prevalence of *Ascaris*.

## Conclusions

We employed the *As*-Hb antigen-based ELISA assay to measure the seroprevalence of *A. suum* infection/exposure on the fattening pig farms in Sichuan Province, China. To our knowledge, this is the first time that a serological screening test has been applied in China. The result of this ‘snapshot’ investigation indicate that *Ascaris* infection in commercial pig farms in Sichuan Province is seriously underestimated, encouraging future large-scale serological studies in different regions of China for a better understanding of the distribution and extent of *Ascaris* exposure and infection. Such a focus should contribute to an improved approach to the control of porcine ascariasis both in China and many other countries around the world.

## Data Availability

Data supporting the conclusions of this article are included within the article.

## Abbreviations

*As*-Hb: *A. suum* haemoglobin
CI: confidence interval
ELISA: enzyme-linked immunosorbent assay
OD: optical density
ODR: optical density ratio
PBS: phosphate-buffered saline
PBST: phosphate-buffered saline plus 0.5% (v/v/) Tween 20
PBSM: PBS with 5% (w/v) milk powder
SE: standard error
